# Immunogenicity of therapeutic peptide products: bridging the gaps regarding the role of product-related risk factors

**DOI:** 10.3389/fimmu.2025.1608401

**Published:** 2025-06-18

**Authors:** Montserrat Puig, Sophie Shubow

**Affiliations:** ^1^ Office of Pharmaceutical Quality Research, Office of Pharmaceutical Quality, Center for Drug Evaluation and Research, US Food and Drug Administration, Silver Spring, MD, United States; ^2^ Office of Clinical Pharmacology, Office of Translational Sciences; Center for Drug Evaluation and Research, US Food and Drug Administration, Silver Spring, MD, United States

**Keywords:** immunogenicity, therapeutic peptides, risk assessment, impurities, qualification threshold

## Abstract

The global market for therapeutic peptides is projected to continue to grow at a fast pace in the coming years in response to high demand for these products. The increasing complexity of chemical and recombinant peptide manufacturing processes may impact product quality attributes, including as related to immunogenicity risk. While it is well established that product-related factors, including impurities, can impact the immunogenicity of a biologic product, assessing the actual impact of a specific product quality attribute on immunogenicity is difficult. Despite significant advances in the analytical characterization of complex peptide products, gaps still exist in our understanding of the significance of impurities to the overall peptide immunogenicity risk, and questions remain about what the best-suited control strategies are. These gaps have the largest impact on the assessment of immunogenicity risk of follow-on therapeutic peptide products, when clinical data are not available to inform that risk. Current regulatory guidance on impurity qualification thresholds is sparse, and *in vitro* and in silico immunogenicity assessment methods for evaluating the immunogenicity risk of impurities present technical and methodological limitations. We highlight these challenges and offer points to consider for handling them.

## 1 Introduction

The global market for therapeutic peptides has grown substantially over the past decade and is projected to reach US$ 86.9 billion in 2032 (1). Since the commercialization of the first insulin product in 1923, the global therapeutic peptide sector has expanded to include over 100 products encompassing hormone analogs, growth factors, neurotransmitters and anti-infectives (2–5). For some of the most recently introduced therapeutic peptide products, patent expiration, increasing demand and shortages are expected to bolster the follow-on peptide markets. This commentary identifies scientific gaps that should be addressed for driving regulatory guidance and global harmonization to facilitate broader access to these drugs.

Peptide production methods have evolved over time (6, 7). The first therapeutic peptides were isolated from natural sources (e.g., insulin from animal pancreases). More recently, recombinant technologies and improved chemical synthesis (i.e., solid-phase peptide synthesis (SPPS)) have accelerated the development of therapeutic peptides. Currently, SPPS is industry’s preferred method for the manufacturing of shorter peptide drugs (i.e., those meeting FDA’s statutory definition of “polymers of 40 or fewer amino acids” (8)), often combined with rDNA technology for the manufacture of longer peptides (semi-synthesis). SPPS can be scaled up and is conducive to a wide array of well-controlled chemical modifications designed to enhance the therapeutic performance of peptides. These include site-specific incorporation of unnatural amino acids, cyclization, pegylation, or conjugation (6).

Synthetic peptides are produced via sequential linking of amino acids through a series of chemical reactions, atop a solid support in SPPS (usually a resin). By contrast, recombinant peptides are produced by inserting a DNA sequence encoding the desired peptide into the genome of a host organism (most commonly yeast or bacteria); this process leverages the host cell machinery to translate the DNA sequence into a peptide chain. The increasing complexity of chemical and recombinant peptide manufacturing processes may impact product quality attributes. Understanding this impact is necessary to ensure the quality of peptide drugs and their safety and efficacy. Like therapeutic proteins, some therapeutic peptides can elicit unwanted immune responses.

Evaluation of clinically relevant immunogenicity is an integral part of peptide development programs. Immunogenicity results from an interplay between product, patient, and treatment-related factors (9, 10). The clinical consequences of this interplay are typically evaluated by measuring anti-drug antibody (ADA) responses in the clinical phase of the development program. For products that share significant characteristics (e.g., an active ingredient) with a previously approved peptide drug product, clinical studies designed to establish efficacy and/or safety may not be necessary, depending on the magnitude of the differences with the previously approved product. Manufacturers submitting applications for generic and follow-on peptide products under the 505(j) and 505(b)(2) pathways, respectively (11, 12), in addition to post-approval changes during product lifecycle management (e.g., manufacturing changes (13)), may rely on FDA’s determination that the approved product is safe and effective, based on data submitted in the approved application (including immunogenicity data) (14).

Reliance on safety and efficacy data from a previously approved product is conditioned upon the bridging of differences between that product and the proposed product. In the context of a manufacturing change, follow-on product, or generic product, differences in impurities need to be bridged, as these differences may increase the immunogenicity risk of the proposed product above that of the product it is compared to. For prior approval supplements (PAS) and 505(b)(2) products, this bridge can be established using comparative clinical studies (15). For generic products under abbreviated new drug applications (ANDA), this bridge is established via non-clinical approaches, including robust comparative analytical methods (16).

Despite significant advances in the analytical characterization of complex peptide drug products, gaps still exist in our understanding of the significance of impurities to the overall immunogenicity risk of a peptide drug product, and questions remain about what the best-suited control strategies are. We highlight these gaps and offer points to consider for addressing them.

## 2 How immunogenicity risk factors are assessed during the peptide drug’s lifecycle

**Figure 1:**
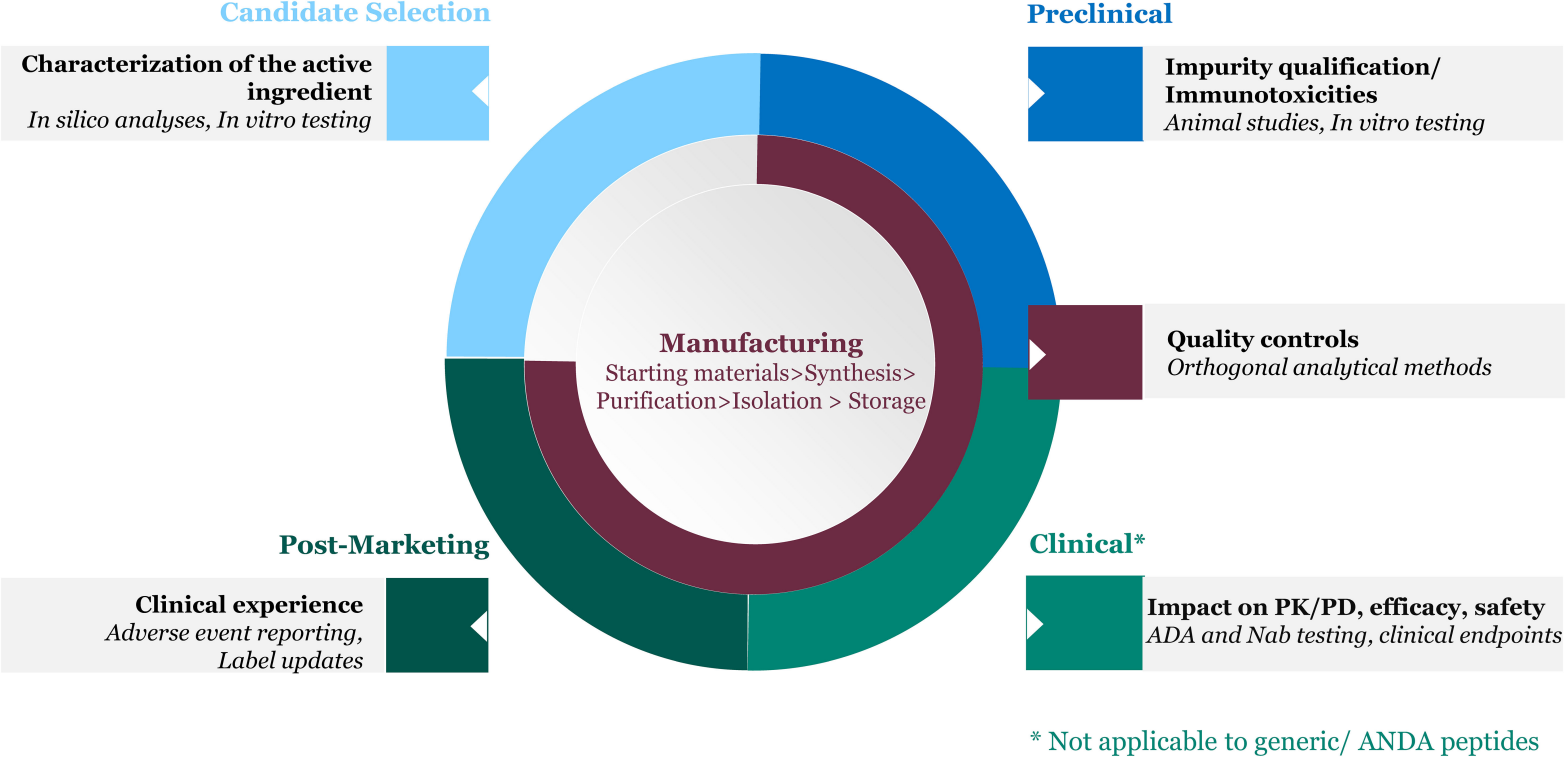
Product factors inform immunogenicity risk assessment throughout the peptide drug lifecycle.

As mentioned earlier, some therapeutic peptides can trigger an unwanted immune response upon administration. The probability of an immunogenic response and potential clinical impact are determined by multiple factors that are characterized and monitored at different stages of the peptide drug’s lifecycle (**Figure 1**).

### 2.1 Candidate selection

An initial step in drug development is selection of an active ingredient amino acid sequence that is therapeutically effective while carrying low risk for immunogenicity. Product-related immunogenicity risk is largely driven by the potential of the active ingredient to stimulate an immune response, based on its origin (i.e., natural sources, synthetic or recombinant) and based on differences in sequence from self-proteins that could be recognized as foreign. Intrinsically, immunogenic epitopes may activate the adaptive immune system and trigger the formation of ADA, and/or activate the innate immune system, leading to hypersensitivity reactions. *In silico* and *in vitro* tools can be leveraged for the selection of peptide sequences with potentially lower immunogenicity (17–19).

### 2.2 Manufacturing

In addition to the active ingredient, which generally represents over 95% or more of the peptide drug by weight, peptide-related impurities resulting from biochemical or biophysical modifications of the active ingredient’s sequence (e.g., insertions, deletions, substitutions, racemization, β-alanine containing contaminants) can be introduced during peptide synthesis (20, 21). These impurities may modulate the overall immunogenicity of the drug product. Recombinant peptide drug products are less likely to contain peptide-related impurities, but more likely to contain process-related impurities derived from the cell-based expression system used to produce the peptide (i.e., DNA, proteins) as well as impurities resulting from post-translational modifications. Both synthetic and recombinant peptides are subject to degradation via mechanisms such as deamidation, oxidation, disulfide bond formation and breakage, to name a few, during manufacturing and storage. Excipients and extractables/leachables from the container closure have the potential to promote higher order structure such as oligomers, fibrils and aggregates, or product degradation (21, 22). Impurities introduced during manufacturing and storage can potentially enhance the immunogenicity of the peptide drug product and may also promote immune-related adverse events. As such, impurity characterization and control throughout the manufacturing process and shelf-life is paramount to ensure consistency in product quality attributes.

### 2.3 Pre-clinical stage

Pre-clinical and toxicology studies in animals can be used to qualify impurities for immunotoxicity i.e., their ability to induce adverse effects on the immune system (immunosuppression or immunoactivation) and may help flag potential clinical impact if a patient were to develop ADA (9, 23).

### 2.4 Clinical stage

Clinical assessment is the definitive way to assess the immunogenicity risk of a product holistically, taking into account all risk factors, and is routinely conducted for innovator peptide drug products. Clinical immunogenicity testing involves developing ADA assays to evaluate impact of ADA on the product’s pharmacokinetics (PK), pharmacodynamics (PD), and efficacy (11), as well as monitoring for relevant safety endpoints such as hypersensitivity reactions. Several risk factors are usually considered in the evaluation of immunogenicity risk: Host-related factors are those associated with the target patient population. Genetics (e.g., HLA, immune repertoire), age, disease, immune status, and co-medications are known to modulate the likelihood and/or magnitude of an unwanted immune response. Treatment-related factors such as delivery route, dose and frequency of drug administration can also impact the product’s immunogenicity risk and are also considered.

### 2.5 Post-marketing stage

Clinical experience accrued in the post-marketing setting can provide further insight into the immunogenicity profile of the product, since clinical studies conducted during development may not be powered to capture rare but potentially serious immune-related adverse events.

## 3 Challenges to assessing product-related immunogenicity risk factors in peptide drug products & implications for follow-on peptide products and manufacturing changes

As mentioned above, in the development phase, a full understanding of a peptide drug product’s clinical immunogenicity profile can only be achieved through clinical studies. However, for the regulatory approval of manufacturing changes and follow-on products under abbreviated pathways, the conduct of clinical studies to establish safety may not be desirable or permissible (in the case of ANDAs). Immunogenicity risk evaluations of these applications rely on an understanding of how product quality differences observed in the new proposed product from the clinically tested reference listed drug could impact the follow-on product’s immunogenicity risk. In this section, we outline regulatory guidance and scientific gaps to be addressed to facilitate insightful immunogenicity risk evaluation of applications without clinical data.

### 3.1 Broadly applicable impurity qualification thresholds are not available for peptide drug products

Although the quantity of impurities in a dose of the drug product is an important determinant of the potential clinical impact of these impurities, there is currently no broadly applicable FDA guidance establishing thresholds for peptide-related impurity identification and qualification. Peptide-related impurity limits and controls are therefore typically determined and justified on a case-by-case basis during product development drawing from manufacturing experience, by batch and stability data, and toxicology data.

Recombinant peptide drug products are in scope of ICH Q6B, issued by the International Conference on Harmonization of Technical Requirements for Pharmaceuticals for Human Use (ICH). This document provides guidance on the setting and justification of product quality specifications, including those related to impurities, but does not recommend specific test procedures or acceptance criteria (24) (**Table 1**).

**Table 1:** Guidance covering peptide product quality applicable in the US.

Guidance	Scope	Impurity qualification threshold	Basis for impurity qualification threshold	Impurity qualification method(s)
ICH Q6B	Recombinant peptides (standalone and follow-ons)	None recommended	Not applicable	None recommended
ICH Q3A/ Q3B	Chemically synthesized molecules – excluding peptides	Threshold calculated based on the maximum daily dose (MDD). For an MDD≤2g/day: 0.15% of DS (or 1.0 mg/day) or lower if impurity known to be unusually toxic	Toxicity	Case-by-case. No specific focus on immunogenicity: Toxicology studies: genotoxicity studies, general toxicity studies, other toxicity studies
2021 Peptide ANDA guidance (limited scope)*	Generic synthetic peptides	New impurities: 0.10%-0.5% of DS Common Impurities > RLD	Unspecified impurities in finished peptide products (0.5%)	In Vitro/ In Silico Immunogenicity Assessments
ICH Q5E	Recombinant peptides (manufacturing changes)	None recommended	Not applicable	Case-by-case basis; “The extent and nature of nonclinical and clinical studies will be determined on a caseby-case basis in consideration of various factors, which include among others: [Drug product]: The type, nature, and extent of differences between the post-change product and the pre-change product with respect to quality attributes including product-related substances, the impurity profile, stability, and excipients. For example, new impurities could warrant toxicological studies for qualification”

*Applicability limited to products referencing five RLDs and select number of additional products per product specific guidance documents.

For standalone synthetic peptides, the principles of ICH Q3A and ICH Q3B may be considered applicable (**Table 1**); these guidance documents provide thresholds for the reporting, identification and qualification of impurities in chemically synthesized new drug substances and new drug products respectively (25, 26). The only guidance explicitly covering synthetic peptide drug products is EMA’s 2023 draft guideline (27), which recommends compliance with the general monograph Ph. Eur. 2034 (28). The United States Pharmacopeia (USP) General Chapter <1503> addresses quality considerations for synthetic peptide drug substances but does not recommend impurity limits (29). Peptide-related impurity limits and controls are determined and justified on a case-by-case basis during product development drawing from manufacturing experience, by batch and stability data, and toxicology data.

#### 3.1.1 Implications for comparative immunogenicity evaluations

The lack of qualification limits for peptide-related impurities impacts the evaluation of impurity differences between a 505(b)(2) or generic peptide and a listed drug/reference listed drug, and between pre- and post-manufacturing change products during product lifecycle. The central question is the extent to which differences in impurity profiles and quantities need to be controlled to ensure comparability in terms of immunogenicity risk.

In 2021, FDA finalized guidance (“ANDAs for Certain Highly Purified Synthetic Peptide Drug Products That Refer to Listed Drugs of rDNA Origin”, hereby referred to as “2021 Peptide ANDA guidance”) to address this question (16). For the five products in its scope, the guidance ties immunogenicity risk to specific differences in impurity content between the proposed generic peptide product and its reference listed drug (RLD) for (1) peptide-related impurities common to the generic product and the RLD, when these impurities are measured at levels higher than those found in the RLD; and (2) new impurities (detected solely in the generic product), when those impurities are measured between 0.10% and 0.5% of the drug substance (**Table 1**). The guidance recommends that the risk associated with these impurities be assessed using *in vitro* and in silico immunogenicity assessment (IVISIA) methods, discussed in more detail below. Because of the scope of the 2021 Peptide ANDA guidance, the applicability of this guidance to ANDA peptide products referencing other RLDs is assessed on a product class-basis and captured in product specific guidance documents when a new RLD has become available (30).

The lack of broadly applicable impurity qualification thresholds also affects the assessment of the immunogenicity risk associated with manufacturing changes. Major changes in the manufacturing process for 505(b)(1) peptide products are submitted for FDA approval via a prior approval supplement (PAS)(13). A PAS is deemed acceptable when the data presented in the supplement demonstrates that the proposed change(s) do not adversely impact the product’s quality, safety and/or efficacy.

For peptides derived from biological sources, ICH Q5E (15) articulates general principles for manufacturers to consider when they assess the risk associated with a manufacturing change, including the risk to a product’s immunogenicity profile (**Table 1**). In the case of a PAS submitted for a manufacturing change, comparability can often be established through a comparison of critical quality attributes of the pre- and post-change product, provided that the pre-change product is well-characterized, and the impact of the process change on critical quality attributes is adequately assessed. ICH Q5E recommends considering both the probability that the change may impact immunogenicity and the consequences of the potential impact to determine whether clinical bridging studies are needed beyond quality information. For manufacturing changes affecting synthetic peptides, while no guidance exists, the potential impact of a proposed change on the product’s safety and efficacy is expected to be evaluated.

### 3.2 When available, impurity qualification thresholds are not informed by immunogenicity risk

ICH Q3A and ICH Q3B tie qualification thresholds for impurities found in the drug substance (ICH Q3A) and product-related impurities in the drug product resulting from degradation of the drug substance (ICH Q3B) to toxicology considerations, adjusting thresholds based on the maximum daily dose of the drug product. The recommended thresholds are based on genotoxicity studies and other toxicity studies, which are the types of studies recommended to qualify impurities present at levels exceeding the recommended qualification threshold.

#### 3.2.1 Implications for comparative immunogenicity evaluations

The 2021 Peptide ANDA guidance states that any new peptide-related impurity present at 0.10%-0.5% of the drug substance should be characterized for immunogenicity risk. The guidance document explains that the 0.5% upper limit “is consistent with the small amount of unspecified peptide-related impurities observed in finished peptide products”, per product knowledge accrued at the time.

There is no available guidance currently that specifies the magnitude of differences in quality attributes between two products that would allow to consider these products comparable in terms of immunogenicity risk.

### 3.3 Challenges associated with IVISIA methods for comparative immunogenicity evaluations

It is well-established that non-clinical and IVISIA data do not predict clinical immunogenicity (31). However, these methods provide a means of assessing differences in immune activation by product quality attributes, namely impurities, that differ between a follow-on product and a reference listed drug. As stated earlier, clinical immunogenicity is multi-factorial, impurities being one of the risk factors. Non-clinical and IVISIA methods are not designed to model the complexity of a potential immune response in humans. However, although a signal detected by these methods does not directly translate into clinical relevance, it may alert the manufacturer to the need to further purify their product.

Below is an overview of IVISIA methods that highlights the potential of these methods and their current limitations, which were discussed in a recent public workshop co-hosted by FDA and the Center for Research on Complex Generics (32).

#### 3.3.1 *In silico* tools

In silico algorithms are cost-effective tools for rapidly assessing the immunogenic potential of impurities, allowing for the screening of numerous peptide-related impurities for epitope sequences that may bind to HLA and result in T-cell activation or antibody formation. However, they have several limitations: (1) like other IVISIA methods, *in silico* algorithms cannot fully assess clinical immunogenicity, (2) their accuracy in evaluating HLA binding is contingent on the algorithm and training dataset, and (3) peptides with unnatural or stereoisomeric amino acid residues or modified side chains cannot be evaluated. Therefore, *in silico* tools cannot be used in isolation, and experimental validation of in silico analyses is necessary.

#### 3.3.2 *In vitro* immunogenicity assays

Different types of *in vitro* assays can offer a broad understanding of impurity presentation and activation of immune cells. Cell-based assays that evaluate T-cell dependent immune responses to the drug product have long been used in pre-clinical immunogenicity risk assessments for therapeutic proteins (33). Use of these *in vitro* immunogenicity assays as regulatory tools to qualify peptide-related impurities, however, present significant technical challenges. *In vitro* cell-based methods use human immune cells (e.g. PBMC or DC:T-cell) and evaluate markers of immune activation such as cytokine production and T-cell proliferation in response to an impurity of concern. However, the low number of naive T-cells circulating in the blood and low number of immunogenic epitopes in peptide sequences limit the sensitivity of these assays. Moreover, excipients in the drug product formulation may impact cell viability and function, which makes it difficult to assess immune cell activation by a formulated drug product. Lastly, cell-based assays are not sufficiently sensitive to inform immune responses against the quantity of impurities found in the drug product. Due to this limitation, impurities that require immunogenicity assessment are generally individually synthesized, purified, and tested at concentrations far exceeding their concentration in the drug product.

PBMC and cell-line-based assays are also used to conduct comparative innate immunity studies and evaluate the risk associated with differences in non-peptide process-related impurities and aggregates between a test product and a reference listed drug. Use of *in vitro* immunogenicity assays as regulatory tools, however, present challenges. As with the adaptive immune assays, certain excipients may interfere with assay readout (34, 35).

Overall, use of *in vitro* immune assays for regulatory purposes would benefit from availability of validated controls and standardized experimental conditions, as discussed at the 2024 FDA-CRCG workshop (32).

#### 3.3.3 *Ex vivo* and *in vivo* models

Systems such as tissue explants, microphysiological systems (e.g., organoids or organ-on-a-chip), and transgenic animal models, have been proposed to analyze a more comprehensive set of components of the clinical immune response to therapeutic proteins (36–39). In general, animal studies are not considered predictive of human immunogenicity due to interspecies differences in metabolism, MHC molecules, and immune receptors. However, animal-based or other toxicological studies may inform of the immune toxicities associated with peptide impurities. Nevertheless, animal studies for the sole purpose of evaluating impurities are discouraged, particularly in light of recent focus on alternatives to animal testing (e.g., new approach methodologies) and the FDA Modernization Act 3.0 (40).

## 4 Points to consider when assessing impurity-level immunogenicity risk

While providing a path for the approval of complex generic peptide products, the 2021 Peptide ANDA guidance’s stated scope is limited to ANDAs referencing five recombinant RLDs associated with different levels of clinical immunogenicity, which means that its applicability to additional ANDA peptides is assessed *de novo* on a product-class basis. Of note, the guidance does not cover 505(b) (2) peptide products, some of which may have minimal differences to the listed drug and, as such, be very similar to ANDA products in terms of immunogenicity risk. Below are points to consider when handling uncertainties around the potential clinical risk of new impurities or differences in impurity levels until more broadly applicable guidance becomes available. Some of these points apply not only to follow-on peptides but also to innovator peptides.

### 4.1 Thorough analytical characterization of the product can help target immunogenicity testing to impurities that need to be assessed

Assessing immunogenicity of impurities early in the development program may seem beneficial. However, these assessments cannot be properly conducted without aged batches or long-term stability data, since those will provide degradation impurity levels at or close to, the end of product shelf-life that cannot be predicted with younger batches. A stepwise approach where only impurities that cannot be controlled at levels consistent with the RLD are tested for immunogenicity risk, will limit the need for testing.

### 4.2 The risk associated with impurity differences should be contextualized

Impurity differences between two products expected to be comparable may translate into variations in immunogenicity. However, the magnitude of the impact of differences in impurity profiles should be evaluated in the context of all the factors influencing the product’s clinical immunogenicity profile. Insights from published research on the risk associated with certain classes of impurities, including aggregates and particulates (41), as evaluated in *in vivo* systems can help assess the level of uncertainty and determine the need for mitigation strategies.

### 4.3 Open-source repositories for peptide-related impurities could benefit both industry and regulators

Drug developers can benefit from sharing non-clinical and clinical data that inform the immunogenicity risk associated with impurities, and ultimately making this data publicly accessible, using consortium approaches, as exemplified by databases that categorize host cell proteins in recombinant peptide products according to their risk to product quality and patient safety (42).

### 4.4 Research can inform impurity thresholds with potential to induce an immune response to peptide drug products

By the end of the purification process, individual peptide-related impurities are present at a very low proportion in the drug product compared to the active ingredient. Evaluating the immunogenicity risk associated with an impurity present in such low quantity, is difficult. Risk assessment strategies in support of peptide impurity safety have been recently proposed (43). Moreover, well thought-out experimental approaches may also provide a path to establish the lowest quantity of impurity detectable by a given *in vitro* immunogenicity assay. Knowledge about what the lowest quantity of peptide drug impurities is that could lead to an immune response, is needed to establish qualification thresholds. Experimentally derived qualification thresholds could then be confirmed by cross-referencing the impurity levels reported in therapeutic peptide products.

### 4.5 The integrated summary of immunogenicity can facilitate immunogenicity risk management and regulatory review of peptide drug products

The integrated summary of immunogenicity (ISI) provides a template for the structured presentation of all the information pertaining to the immunogenicity risk of a proposed product. Importantly, the format of the ISI links the analysis of risk factors to the justification of the approach taken for risk evaluation and mitigation, and to immunogenicity results. As such, the ISI supports the tailoring of immunogenicity assessment and mitigation strategies to the specific immunogenicity risk of the product. This document can be conceived as a living document that can be created as early as candidate selection stage, and updated as new data are generated, enabling an agile regulatory review of immunogenicity across disciplines. While broadly adopted for biological products, as recommended by regulatory agencies (27, 44–46), this document is not typically found in regulatory submissions for therapeutic peptide products but could provide notable value.

## 5 Conclusion

While it is established that product-related factors can impact the immunogenicity of a biologic product, assessing the actual impact of a specific product quality attribute on immunogenicity is difficult, whether in clinical trials or in the post-marketing setting. Quality data from clinical lots can be correlated with ADA information generated during clinical studies, but conclusions will be confounded by patient- and treatment-related factors. Qualification thresholds for impurities provided in guidance applicable to peptide drug products, when available, are determined based on toxicity considerations or limited experience with products available commercially. Recommended qualification strategies involve toxicity studies as well as *in vitro* and in silico immunogenicity assessment (IVISIA) methods. The usefulness of these methods will increase as current technical and methodological limitations are addressed. Considering the current challenges, there is value in prioritizing impurity control strategies and drawing from available information on the immunogenicity risk of specific impurities (e.g., databases, literature). Lastly, the risk associated with a particular impurity is better understood in the context of the overall immunogenicity risk of the product, which an ISI can help capture. Taken in combination, these approaches can help mitigate the risk associated with impurities until updated impurity qualification thresholds informed by immunogenicity risk are available.

## Data Availability

The original contributions presented in the study are included in the article/supplementary material. Further inquiries can be directed to the corresponding author/s.
